# ASSOCIATION BETWEEN PET OWNERS’ AND PETS’ PHYSICAL ACTIVITY LEVELS: A SYSTEMATIC REVIEW

**DOI:** 10.1093/geroni/igad104.2005

**Published:** 2023-12-21

**Authors:** Nanna Notthoff

**Affiliations:** Leipzig University, Leipzig, Sachsen, Germany

## Abstract

Social support can promote physical activity (PA) participation. According to socioemotional selectivity theory, engaging in PA with or for important social partners may be particularly motivating for older adults. Recently, pets have been recognized as important social partners. Although studies and literature syntheses have compared PA levels of people with vs. without pets, less is known about whether humans and pets are actually active together, i.e., whether their activity levels are associated. We examined this question in a systematic literature review. Through searches in Web of Science and PubMed with a priori specified inclusion and exclusion criteria, we identified 17,565 potential records. A two-step screening process yielded 9 studies for inclusion in the systematic review. Using a modified version of the STROBE checklist, we rated their quality as M = 21.89 (SD = 2.93) out of 30 possible points. Of the 9 included studies, only 5 explicitly calculated the association between human and pet PA; 4 found a positive association and 1 found no association. Of the 9 included studies, 4 studies explicitly included older adults 60+ years. However, variations in the association between human and pet PA by age were not examined. It seems that humans and their pets are active together. A more thorough examination of this question could provide a basis for an intervention aimed at increasing human PA by incorporating pets. Such an intervention may be particularly suitable for older adults who have lost important human social partners or who have difficulty accessing PA group programs.

